# Adesão à Vacina Contra Influenza em Pessoas Idosas – Reflexões e Perspectivas

**DOI:** 10.36660/abc.20250210

**Published:** 2025-05-29

**Authors:** Bruna Moretti Luchesi

**Affiliations:** 1 Universidade Federal de Mato Grosso do Sul Campo Grande MS Brasil Universidade Federal de Mato Grosso do Sul (UFMS), Campo Grande, MS – Brasil

**Keywords:** Cobertura Vacinal, Doenças Cardiovasculares, Idoso, Vacinas contra Influenza

A vacinação contra influenza no Brasil foi implantada em 1999 com o objetivo de vacinar pelo menos 70% da população com 65 anos ou mais. Em 2000, a idade baixou para 60 anos; em 2010, novos grupos de risco foram incluídos como elegíveis a receber a vacina; em 2008 a meta de vacinação subiu para 80%; e em 2017 para 90%,^1^ a qual se mantém até os dias atuais.

As pessoas idosas são consideradas um grupo de risco para vacinação por apresentarem maior suscetibilidade a doenças graves e complicações da influenza.^2^ Dentro desse grupo, aqueles que possuem comorbidades cardiovasculares são ainda mais vulneráveis.^3^

Dados do Sistema de Informações do Programa Nacional de Imunizações indicam que a meta vacinal de 90% foi atingida no grupo de pessoas idosas de 2016 a 2019; e que, em 2020, a cobertura vacinal chegou a 120,7%.^4^ No entanto, a partir de 2021, os dados mostraram uma queda na cobertura vacinal para influenza nesse grupo, chegando a 70,9% e continuando a cair nos anos seguintes: 2022 (70,2%), 2023 (63,3%) e 2024 (48,7%).^5^ Assim, evidências atuais sobre a adesão vacinal e motivos para não adesão se mostram necessárias em nosso país.

O estudo de Aguilar et al.,^6^ teve como objetivo analisar a adesão à vacinação contra influenza em pessoas idosas com comorbidades cardiovasculares, utilizando dados de 5.296 indivíduos, coletados na segunda onda do projeto ELSI-Brasil (2019-2021).

Trata-se de uma investigação com amostra grande e representativa, que identificou cobertura vacinal de 76,6% entre os participantes.^6^ A média de cobertura vacinal entre 2019 e 2021 no Brasil foi 96,99%,^5^ indicando que os resultados encontrados estão abaixo da cobertura vacinal do país. Um dos motivos para essa inconsistência pode ser a superestimação das coberturas vacinais, que aconteceu até o ano de 2020, devido à desatualização do número de pessoas idosas utilizadas no denominador do cálculo da cobertura, como já reconhecido anteriormente.^7^ Além disso, o fato de o estudo incluir apenas pessoas idosas com comorbidades cardiovasculares pode ser um fator importante, já publicações anteriores mostram que ter comorbidades pode ou não aumentar a adesão à vacina contra influenza.^8 , 9^

Entre os fatores associados a uma maior adesão à vacina, foram elencados maior idade, estar casado ou vivendo com companheiro, fazer uso de serviços de saúde privados e ter autopercepção de saúde boa ou excelente. Já os fatores ser da etnia negra, consumir álcool mais de uma vez ao mês, ser tabagista e praticar atividades físicas intensas mais de uma vez por semana foram relacionados à menor chance de adesão à vacina.^6^ Esses dados são consoantes à estudos anteriores^8 , 9^ e são excelentes indícios dos grupos mais vulneráveis à não adesão vacinal, os quais devem ser alvo de intervenções.

Além disso, também foi conduzida uma análise de acordo com cada comorbidade cardiovascular estudada (i.e., hipertensão, angina, infarto e insuficiência cardíaca). A idade permaneceu significativa para todas as comorbidades. Para hipertensão, identificou-se também como fatores etnia preta, consumo de álcool mais de uma vez ao mês, tabagismo, prática de atividades físicas intensas mais de uma vez por semana e melhor autopercepção da saúde. Para insuficiência cardíaca, o outro fator significativo foi o tabagismo; e para angina e infarto do miocárdio, não foram identificados fatores adicionais.^6^ Assim, os fatores podem ser diferente em cada grupo de doenças, indicando a necessidade de uma maior atenção dos profissionais de saúde.

O estudo aborda ainda os motivos para não adesão à vacina, com destaque para questões pessoais como medo de reações adversas, raramente contrair gripe e contraindicação médica; seguidas pelas questões contextuais como indisponibilidade da vacina; e questões específicas da vacina, como não saber que era necessário tomar a vacina contra gripe e não acreditar que a vacina ofereça proteção contra a doença.^6^ Alguns motivos chamam a atenção e já haviam sido relatados anterioremente,^10^ como a falta de orientação sobre a necessidade e importância da vacina, evidenciando o papel crucial dos profissionais de saúde; bem como a indisponibilidade da vacina, que indica a necessidade de uma maior organização do sistema de saúde para atender às demandas.

As pessoas idosas compreendem um grupo que, no geral, possui baixo letramento em saúde.^11^ Por esse motivo os esforços para promoção da saúde, especialmente sobre a questão vacinal, nesse grupo, devem ser redobrados. Destaca-se a importância dos profissionais da saúde no combate às notícias falsas sobre a vacinação para que a alta cobertura vacinal, observada no país desde o início da campanha até o ano de 2020, possa ser alcançada novamente,^12^ evitando assim que casos graves e óbitos preveníveis causados pelo vírus influenza ocorram. Estaremos vivenciando mais um ano de queda nas coberturas vacinais contra influenza? Qual é o nosso papel nesse cenário?
