# Supplementary material for: Adesão à Vacina Contra Influenza em Pessoas Idosas – Reflexões e Perspectivas
Source: Arq Bras Cardiol. 2025 May 29;122(3):e20250210. [Article in Portuguese] doi: 10.36660/abc.20250210 (PMC12212372; doi:10.36660/abc.20250210)
Supplement: Translation

# Translation: Influenza Vaccine Adherence in Older Adults – Reflections and Perspectives

**DOI:** 10.36660/abc.20250210i

**Authors:** Bruna Moretti Luchesi

**Affiliations:** 1 Universidade Federal de Mato Grosso do Sul (UFMS) Campo Grande MS Brazil Universidade Federal de Mato Grosso do Sul (UFMS), Campo Grande, MS – Brazil

**Keywords:** Vaccination Coverage, Cardiovascular Diseases, Aged, Influenza Vaccines

Influenza vaccination in Brazil was implemented in 1999 with the goal of vaccinating at least 70% of the population aged 65 or over. In 2000, the age limit was lowered to 60 years; in 2010, new risk groups were included as eligible to receive the vaccine; in 2008, the vaccination target was raised to 80%; and in 2017, to 90%,^1^a target that remains to this day.

Older people are considered a risk group for vaccination because they are more susceptible to serious illnesses and complications from influenza.^2^Within this group, those with cardiovascular comorbidities are even more vulnerable.^3^

Data from the National Immunization Program Information System indicate that the 90% vaccination target was reached in the elderly group from 2016 to 2019 and that, in 2020, vaccination coverage reached 120.7%.^4^However, as of 2021, the data showed a drop in influenza vaccination coverage in this group, reaching 70.9% and continuing to fall in the following years: 2022 (70.2%), 2023 (63.3%) and 2024 (48.7%).^5^Thus, current evidence on vaccination adherence and reasons for non-adherence is necessary in our country.

The study by Aguilar et al.^6^aimed to analyze adherence to influenza vaccination in older adults with cardiovascular comorbidities, using data from 5,296 individuals collected in the second wave of the ELSI-Brazil project (2019-2021).

This is a study with a large and representative sample, which identified vaccination coverage of 76.6% among participants.^6^The average vaccination coverage between 2019 and 2021 in Brazil was 96.99%,^5^indicating that the results found are below the country's vaccination coverage. One of the reasons for this inconsistency may be the overestimation of vaccination coverage, which occurred until 2020, due to the outdated number of older adults used in the denominator of the coverage calculation, as previously recognized.^7^In addition, the fact that the study included only older people with cardiovascular comorbidities may be an important factor since previous publications show that having comorbidities may or may not increase adherence to the influenza vaccine.^8 , 9^

Factors associated with greater vaccine adherence included being older, being married or living with a partner, using private health services, and having a self-perceived good or excellent health. Factors such as being black, consuming alcohol more than once a month, being a smoker, and engaging in intense physical activity more than once a week were associated with a lower chance of vaccine adherence.^6^These data are consistent with previous studies.^8 , 9^They are excellent indicators of the groups most vulnerable to vaccine non-adherence and which interventions should target.

In addition, an analysis was also conducted according to each cardiovascular comorbidity studied (i.e., hypertension, angina, infarction, and heart failure). Age remained significant for all comorbidities. For hypertension, black ethnicity, alcohol consumption more than once a month, smoking, intense physical activity more than once a week, and better self-perception of health were also identified as factors. For heart failure, the other significant factor was smoking, and for angina and myocardial infarction, no additional factors were identified.^6^Thus, the factors may be different in each group of diseases, indicating the need for greater attention from health professionals.

The study also addresses the reasons for non-adherence to the vaccine, highlighting personal issues such as fear of adverse reactions, rarely contracting the flu, and medical contraindication, followed by contextual issues such as unavailability of the vaccine and specific issues related to the vaccine, such as not knowing that it was necessary to take the flu vaccine and not believing that the vaccine offers protection against the disease.^6^Some reasons are noteworthy and have already been reported previously,^10^such as the lack of guidance on the need for and importance of the vaccine, highlighting the crucial role of health professionals, as well as the unavailability of the vaccine, which indicates the need for greater organization of the health system to meet demands.

Older adults are a group that generally has low health literacy.^11^For this reason, efforts to promote health, especially regarding vaccination, in this group must be redoubled. It is important to highlight the importance of health professionals in combating fake news about vaccination so that the high vaccination coverage observed in the country from the beginning of the campaign until 2020 can be achieved again,^12^thus preventing serious cases and preventable deaths caused by the influenza virus from occurring. Will we be experiencing another year of declining vaccination coverage against influenza? What is our role in this scenario?
